# “How Am I Going to Do It?” Understanding the Challenges of Assuming Care of a Child Born During their Mothers’ Incarceration

**DOI:** 10.1089/heq.2024.0098

**Published:** 2024-10-09

**Authors:** Bethany Kotlar, Kate Dawson, Varshini Odayar, Ellen Mason, Henning Tiemeier

**Affiliations:** ^1^ Harvard Graduate School of Arts and Sciences, Boston, Massachusetts, USA.; ^2^ Rollins School of Public Health, Emory University, Atlanta, Georgia, USA.; ^3^ Harvard Medical School, Boston, Massachusetts, USA.; ^4^ University of Illinois Chicago, Chicago, Illinois, USA.; ^5^ Harvard T.H. Chan School of Public Health, Boston, Massachusetts, USA.

**Keywords:** incarceration, infant health, family health, qualitative research

## Abstract

**Objectives:**

Mass incarceration of women systematically targets minoritized groups. Approximately 1,000 births occur from prison annually; and most children must be cared for by someone other than their mother. Little is known about caregiving for infants in the context of maternal incarceration. The purpose of this study was to describe the experiences of caregivers assuming care of newborns of incarcerated mothers to identify challenges and provide policy recommendations.

**Materials and Methods:**

Data from this study were drawn from qualitative intake interviews with caregivers of children born to incarcerated mothers in Georgia participating in the Birth Beyond Bars Study, an ongoing birth cohort of children exposed prenatally to incarceration in three states. One of the primary research questions for these interviews was how caregivers were coping with assuming care for the infant. All caregivers of children born to incarcerated women from August 2020 to January 2023 (*n* = 48) were approached for enrollment into the study by staff at a nonprofit providing support to incarcerated pregnant and postpartum women and their families. We used thematic analysis to analyze data from 36 caregivers.

**Results:**

Caregivers were primarily older (45% were between 46 and 71), female kin (89%), who were frequently impoverished (30%). Caregivers faced challenges in legally assuming care of the infant, maintaining work, securing childcare, enrolling in social services, and managing their health. They primarily relied on their families for support.

**Discussion:**

Caregivers of infants of incarcerated mothers are a vulnerable population requiring enhanced support. Targeted support may ameliorate negative consequences of assuming this role.

**Health Equity Implications:**

To promote health equity, state social service agencies should target and assist caregivers in enrolling in social services. Most importantly, states should pass legislation prioritizing community-based alternatives to incarceration for pregnant and parenting mothers.

## Introduction

The United States has the highest women’s incarceration rate in the world.^1^ Mass incarceration of women systematically targets marginalized racial groups and the poor.^2–4^ Incarcerated women are also primarily of reproductive age;^5^ an estimated 4% of women are pregnant at their admission into state prisons, and approximately 1,000 women give birth from prison each year.^6^ Those that give birth during their incarceration typically must designate a caregiver for their infant, as few states have nursery programs or community-based alternatives to incarceration.^7,8^ While few studies have investigated the characteristics of caregivers of children born during their mothers’ incarceration, studies of older children of incarcerated women have found stark differences between caregivers of children of incarcerated mothers and those of incarcerated fathers. Children with incarcerated fathers are typically cared for by their mothers, whereas most children with incarcerated mothers are cared for by grandparents or other relatives.^9,10^

Caring for a child of an incarcerated woman may worsen the health and well-being of caregivers. There is evidence that families of incarcerated mothers are negatively impacted by her incarceration through loss of her income,^11^ potential loss of child support,^12^ and the significant cost of legal fines and fees and maintaining contact with an incarcerated mother.^13,14^

Assuming care for a newborn may have an even greater impact on caregivers given the intensive financial and physical demands they entail. Caregivers of newborns must source specialized, typically expensive infant care supplies such as a car seat, strollers, diapers, and formula. In addition, they need to arrange childcare or take, often unpaid, time away from work. Finally, the physical demands of caring for a newborn include frequent sleep deprivation and lifting activities, which may be particularly difficult for older caregivers. In one of the few studies on caregiving for children born during their mothers’ incarceration Pendleton et al. retrospectively asked caregivers, primarily grandparents, to describe challenges of assuming care for children.^14^ Caregivers reported loss of income and worsening physical and mental health due to their caregiving responsibilities, financial issues due to difficulty maintaining a job considering lack of childcare, and difficulty enrolling in social support services. However, at the time of their research, only 45% of caregivers-maintained custody of the child, which may have influenced their narratives regarding caregiving.^14^ Thus, no studies have contemporaneously documented the impact of assuming care for newborns of incarcerated mothers on caregivers, despite the potential heightened difficulty of care in early infancy.

Although caregivers may be negatively impacted by their caregiving role, they can be an important factor in the health and development of children of incarcerated women.^15,16^ Infancy is a unique, sensitive period in child development in which multiple systems are rapidly maturing.^17^ Yet, there are no studies that have linked caregiving arrangements to developmental outcomes for children born during their mothers’ incarceration outside of a prison nursery setting.^18^ There is, however, evidence that caregiving arrangements are associated with developmental outcomes in older children. In a sample of 60 children between the ages of two and seven experiencing maternal incarceration, Poehlmann found that caregiver sociodemographic risk factors such as poor health, lower educational attainment, and higher number of dependents positively predicted lower cognitive scores, but this relationship was mediated by a warm and supportive family environment.^19^ Caregivers’ relationship to the child is also important in maintaining mother/child contact during her incarceration. For older children, grandparental care in the context of maternal incarceration is associated with more frequent contact between children and their mother as well as an increased likelihood of their reunification on her release,^20,21^ however, it is unclear whether this association is true in the context of maternal incarceration at birth.

Given the demonstrated consequences of caregiving during maternal incarceration on caregivers, mothers, and children, the unique challenge of caregiving during the newborn period, and the importance of infancy for later developmental outcomes, it is imperative to understand the unique struggles of caregivers of children born during their mothers’ incarceration. The purpose of this study was to better understand the experiences of caregivers of newborns of incarcerated mothers to identify challenges associated with caregiving as well as sources of support to inform both future research with this population and to provide policy recommendations to increase the health equity of this marginalized population.

## Materials and Methods

Data for this study were drawn from semi-structured interview questions posed to participants of the Birth Beyond Bars Study (BBB Study) during intake surveys. The BBB Study is a mixed methods cohort of children exposed prenatally to incarceration in several states. Interviews used for this study were collected as part of a pilot cohort conducted in Georgia. In Georgia, the BBB Study was implemented in collaboration with Motherhood Beyond Bars (MBB), a nonprofit supporting this population. Forty-eight caregivers of infants born to incarcerated mothers were approached by MBB staff for participation in the study. Eleven caregivers declined enrollment. Enrolled participants were interviewed via phone shortly after the birth of the infant; 91% occurred when the infant was three months old or younger.

Semi-structured interview questions were developed by B.K., a researcher with a decade of practice and research experience working with this population, in collaboration with MBB staff and other experts. Questions were revised during data collection as needed. See Table 1 for interview questions. BK trained MBB staff members, who have both professional and lived experience of incarceration, in qualitative interviewing. MBB staff members and BK conducted interviews between August 2020 and January 2023. BK reviewed interview transcripts to ensure data quality. One MBB staff member’s interviews were flagged for poor data quality; they did not interview subsequent participants. Impacted participants were approached to be re-interviewed between May and August 2021. Twelve caregivers were re-interviewed. Interviews lasted between 20 and 60 min and were audio recorded and transcribed verbatim. Data collection was approved by Harvard’s Longwood Medical Area Institutional Review Board (IRB 20–1215, 21–1247).

**Table 1.** Key Semi-Structured Interview Questions

How did your family and friends react to the news that you would be the caregiver for (infant name)?
How did you prepare to receive (infant name)?
How has your life changed since you started caring for (infant name)?
How has caring for (infant name) impacted your relationships?
How has caring for (infant name) impacted your work?
How has caring for (infant name) impacted your health?
What struggles have there been caring for (infant name)?
What did you do to overcome these challenges?
What has been joyful about caring for (infant name)?

### Data analysis

We used thematic analysis as proposed by Braun and Clarke to analyze data.^22^ The analysis was conducted by an analytical team led by B.K. Researchers developed a codebook of inductive and deductive codes through intensive reading of six randomly selected transcripts, weekly team discussion and code application in Dedoose by two members of the research team to 12 in-depth interview transcripts. Members of the team subsequently completed code application tests through Dedoose until a composite .8 Cohen’s kappa was obtained. Each transcript was independently double coded. Coding discrepancies were resolved through team discussion. Codes were then grouped according to the research question and described. Descriptions were read and discussed to develop preliminary themes. Themes were validated through three member-checking focus groups, two with staff from MBB, and one with five caregiver participants. Focus group transcripts were read inductively and integrated into preliminary themes to develop final themes. We followed the consolidated criteria for reporting qualitative research (COREQ) when presenting this study.

## Results

### Participants

Caregiver intake interviews were included until saturation was reached; 36 caregivers were included in the study. Caregivers were primarily women (89%) between 35 and 55 years old (61%); 28% of participants identified as Black and 6% identified as Hispanic. The majority had a high school education or less (67%) and roughly 30% reported living under the Federal Poverty Line. Most caregivers were grandparents of the infant (58%) or other kin (14%). See Table 2.

**Table 2.** Characteristics of Temporary Caregivers Interviewed (*n* = 36)

	Number (%)
Age of the child at first interview	
less than 1 month	18 (50.0)
1–3 months	15 (41.7)
4–6 months	2 (5.6)
7–9 months	1 (2.8)
Age Category (%)	
25–35	7 (19.4)
36–45	13 (36.1)
46–55	9 (25.0)
56–65	6 (16.7)
65+	1 (2.8)
Ethnicity = Hispanic or Latino/a	2 (5.6)
Race = Black	10 (27.8)
Sex = Male	5 (13.9)
Married = Yes	15 (41.7)
Education	
Some high school, no degree	4 (11.1)
GED or alternative credential	5 (13.9)
High school graduate	19 (52.8)
Associate’s degree	4 (11.1)
Bachelor’s degree	1 (2.8)
Master’s or professional degree	2 (5.6)
Missing	1 (2.8)
Work	
Yes	22 (61.1)
Missing	1 (2.8)
Caregiver relationship to child	
Father/Other Parent	4 (11.1)
Foster parent	1 (2.8)
Grandparent	19 (58.3)
Other relative	7 (13.9)
No kinship relationship	5 (13.9)
Living in poverty	
Yes	11 (30.6)
Missing	1 (2.8)

### Themes

#### Receiving the infant’s documents and enrolling in social services

Once the infant was in the custody of the temporary caregiver, the caregiver needed to complete temporary guardianship papers, power of attorney, and take possession of the infant’s social security card and birth certificate. Interviews with caregivers reveal that this process is made difficult by the lack of coordination between the prison and the birthing hospital, which leads to confusion over where and how to complete this paperwork. Other barriers included difficulty getting paperwork notarized, and delays in getting paperwork returned from the prison once it had been sent in. While one participant was able to complete Power of Attorney documents at the hospital, other caregivers were not given that opportunity. Kelly describes her own attempts to complete paperwork:

You’ve got to have some sort of permanency. A person can’t just go and pick up a baby and keep them for years without any paperwork… Do you know that it took me five weeks to get the flipping paperwork that (they) kept returning the paper?… And I said, look, I don’t understand what part of it that you people don’t understand. I can’t do anything. I can’t take him to a doctor. I can’t do anything.

As can be seen above, this lack of formal documentation of temporary guardianship caused significant stress for caregivers and issues for the infant. Several had to delay a pediatrician’s visit or negotiate with a pediatrician to treat the infant without necessary documentation. Caregivers also described difficulty in obtaining the infant’s birth certificate and social security card, which hampered their ability to travel with the infant.

#### Adjusting to caring for the infant

Caregivers described the infant as bringing joy to their household, expressing that the infant “just fits right in,” or is “good for me.” However, caregivers also described assuming care of the infant requiring an adjustment causing stress. Crystal described the shock of receiving a newborn:

When I got home, I was a little overwhelmed because when you’re pregnant and you have a baby, your body tells you what to do, but then when you pick up the baby that somebody else has given birth to, you don’t have that…it was a lot of stress until I got her home and then I felt kind of relief but then I was like, oh, gosh, look at my [sic] baby.

This shock was exacerbated by the uncertainty of how long care was required due to the mother’s legal issues. Brenda said:

The whole ordeal from start ‘til now has been quite a roller coaster. It was almost a relief, I think, to all of us to have it (sentencing) over with, even to her (child’s mother), because there was so much anticipation of what’s going to happen.

Older caregivers often expressed dismay at having to re-enter a stage of life they thought was completed. Carol expressed, “I think the hardest thing is, I done raised mine and sat up all night with colic and then woke up for feedings and stuff. I’m older, I want my rest.” Similarly, Brenda shared her fiancé’s reaction to having to parent newborn Michael right after his own children moved out of the house:

[My] fiancé, he’s got a daughter that’s in her twenties, both my children are in their twenties.…we had the freedom to come and go and do what we wanted. And then with Michael arriving it was kind of like we were restarting again. He was a bit resentful.

Exacerbating the stress of taking in newborn kin was the stigma associated with having an incarcerated relative. Carol shared:

Okay, she’s gonna have this baby, and if she’s gone, how long am I going to have to take care of this baby? Is she going to be better? How am I going to do it? I didn’t expect this in my close to 60 years old that I’d get my grandkids. It’s hard sometimes to be around other people whose life seems to be so perfect and they’ll be talking about their children and how good their daughter is, and the mama’s living a good life and going to school. And then if you are asked, well, you know, I’ve got custody of my grandkids and their mother’s not doing so good. She’s in prison. Then basically people just stop talking to you. They don’t understand it.

Beyond the shock and stigma described above, the most cited stressor for caregivers was sleep deprivation. Robert stated, when asked what the most challenging part of becoming a caregiver to a newborn was, “not sleeping regular. That was really a pain in the ass after 20 years. It was like [every] two hours. Oh my God.”

While some caregivers expressed that their household and extended families were overjoyed by the baby, others reported that the transition was stressful. Brenda’s decision to assume care of the infant caused a temporary separation from her fiancé. Beth described a stressful adjustment for her elderly parents and teenage grandchild. Kelly and Katherine described slightly older children having difficulty adjusting to decreased attention.

#### Physical and mental health

Several caregivers said it was a challenge dealing with their own physical or mental health issues and caregiving. Carol described how her arthritis made carrying a child difficult. Lisa cited her seizure disorder as a source of anxiety related to caring for the baby, as she had difficulty remembering to take her medication. Beth said:

Oh, I suffer. I have arthritis in my knees. I had deformity in my feet. I have a lot of health issues with that and getting around. There was a time that I was on medication, that bothered me because I couldn’t take my medication properly because of the fact that it knocked me out and I was afraid I wouldn’t be able to care for Jackson.

On top of her physical health issues Beth, and another caregiver, Tina, disclosed a history of substance dependence. They described the added stress of being a caregiver as a potential threat to their sobriety and related how their own legal requirements such as attending AA meetings or a requirement to work made caring for the newborn more challenging.

#### Financial difficulties

Caregivers who were working before assuming care of the newborn often had to adjust their work hours or take a leave of absence. Some caregivers delayed going back to work or declined work-related opportunities because of the COVID-19 pandemic. Jasmine, who worked at a nursing facility before becoming a caregiver, told her workplace that she would not be returning because of fear of being infected at work and bringing the infection back home to the infant.

Childcare was often described by caregivers as a “chicken and the egg” kind of problem. Without reliable childcare, they could not work, but without working, they struggled to afford full-time childcare if they did find a job. Brenda related how lack of childcare forced her to cut her hours at work, but that her son, the infant’s father, was working more to help support her and his child. Lisa related her ongoing issues finding reliable childcare:

[Childcare] was the problem. Mine was too young to be [in any] of the daycares around here. Then if I did get her in a daycare, I didn’t have the money to pay for the daycare, ‘cause daycare costs me more than what I was making. And of course I didn’t have CAPS (childcare subsidy) because I didn’t have all the paperwork to get involved with DFACS to get it. If I got regular babysitters, 99% of the time I ended up having to let them go because I found out they were doing stuff they shouldn’t have been doing.

#### Sources of support

In coping with the responsibilities of caring for an infant, caregivers drew on several sources of support, the most frequent being familial support and support from MBB. Caregivers frequently described their immediate and extended families providing financial support, baby supplies, and childcare. Six caregivers lived in intergenerational households and shared caregiving responsibilities with their parents or their adult children. Other families had arrangements in which one member worked and provided financially for the child, while the other provided day-to-day caregiving. Only three caregivers said that they did not have family support.

Friends were also cited as a source of support, frequently in the form of tangible baby supplies or emotional support. Six caregivers reported that their wider communities were able to step in and help. Kelly stated:

My church family brought dinner twice. I have a lady that cooks my food. One of my friends from church paid for my food for the week, which was amazing. People offered to watch my other kids. It’s so nice to see the community, the whole community kind of come together and help take care.

While we did not ask questions about support from other organizations, many caregivers mentioned MBB as a source of support. This included baby supplies; food; and assistance staying connected with the incarcerated mother. Robert succinctly summarized: “Motherhood Beyond Bars is the only support or resources. Once Motherhood Beyond Bars came in, that’s all I needed.”

Social services were rarely mentioned as a helpful resource. Social services were frequently discussed as being stressful, either because the process of enrolling was difficult, or using social services was stigmatizing. Only two caregivers cited social services as helpful. Tina had their food stamp allotment increase when assuming care of the child, and Lisa reported getting needed formula from the WIC office.

## Discussion

This study found that caregivers of children born to incarcerated mothers report several challenges in assuming care of the newborn including maintaining their own health, obtaining the childcare necessary to work, enrolling in social support services, and adjusting to caring for the newborn. In overcoming these challenges, caregivers rarely relied on social support services, and instead leveraged their extended familial and social support networks. Caregivers also reported seeking help from MBB.

These results corroborate findings from Pendleton et al. that caregivers of children born to incarcerated mothers report experiencing stress related to assuming this role, and struggle to maintain their physical and mental health and jobs considering the increased demands of caring for a newborn. Furthermore, similarly to caregivers in Pendleton et al.’s sample, caregivers in Georgia overcome these difficulties by relying on their social support networks. However, Pendleton et al. found that few caregivers reported difficulty enrolling in social services, while caregivers in our sample frequently discussed frustration with the enrollment process. These differences in findings may be due to disparate state social service policies or may reflect the short timeline between interviews for this study and social service enrollment.

Our results represent the first to our knowledge contemporaneous assessment of caregiving in the context of maternal incarceration from birth. As such, they provide valuable evidence to suggest that caregiving for newborns of incarcerated mothers creates unique caregiving difficulties that don’t necessarily arise when assuming care for older children of incarcerated mothers. The key limitation of this study is that we were not able to link these difficulties to child outcomes. More research, particularly longitudinal research, is needed to fully understand the impact of caregiving during infancy on child wellbeing, particularly child development and child welfare involvement.

## Health Equity Implications

Caregivers of infants born to incarcerated mothers, already a marginalized population, face multiple barriers to their health and well-being. To address these barriers and increase the health equity of this population, we propose three key policy and practice interventions. First, our results indicate that caregivers of children born to incarcerated mothers could particularly benefit from access to social support services, including childcare subsidies, Medicaid enrollment, TANF, WIC, SNAP, and financial benefits for grandparents raising grandchildren. Despite the high need for these services, caregivers overwhelmingly described the process of enrolling in social support services as stressful. Relevant state agencies administering these services should specifically target caregivers of children of incarcerated mothers both for eligibility for these programs and to receive support in enrolling themselves and the children they care for in any services for which they qualify. Non-governmental organizations, such as nonprofits and health care organizations that serve marginalized children, should also consider the unique needs of infants of incarcerated mothers and their caregivers when targeting programmatic support and providing care. Finally, the separation of mothers and infants and the need for non-maternal caregivers would be greatly reduced if states adopted policies that prioritized community-based alternatives to incarceration for pregnant and postpartum mothers. Not only would this policy eliminate many of the issues described in this article, it would also promote practices understood to be foundational to maternal and child health, including breastfeeding and early bonding, currently impossible in the status quo of forced maternal separation.

In conclusion, caregivers of infants born during their mothers’ incarceration report significant stress, stigma, and uncertainty in assuming care for these newborns. The difficulties they experience are exacerbated by various institutional barriers. Much can be done in the short term to support these caregivers and increase health equity. In the long term, states must recognize the significant toll that early maternal separation takes on newborns and their families. States should commit to building robust community-based alternatives to incarceration for pregnant incarcerated women.

## Abbreviations Used

BBB: Birth Beyond Bars Study
MBB: Motherhood Beyond Bars
SNAP: Supplemental Nutrition Assistance Program
TANF: Temporary Assistance for Needy Families
WIC: Special Supplemental Nutrition Program for Women, Infants, and Children
